# Amelioration of experimental autoimmune encephalomyelitis by gemfibrozil in mice via PPARβ/δ: implications for multiple sclerosis

**DOI:** 10.3389/fncel.2024.1375531

**Published:** 2024-05-21

**Authors:** Susanta Mondal, Monica Sheinin, Suresh B. Rangasamy, Kalipada Pahan

**Affiliations:** ^1^Department of Neurological Sciences, Rush University Medical Center, Chicago, IL, United States; ^2^Division of Research and Development, Jesse Brown Veterans Affairs Medical Center, Chicago, IL, United States

**Keywords:** gemfibrozil, EAE, MS, blood–brain barrier, neuroinflammation, PPAR, Tregs

## Abstract

It is important to describe effective and non-toxic therapies for multiple sclerosis (MS), an autoimmune demyelinating disease. Experimental autoimmune encephalomyelitis (EAE) is an immune-mediated inflammatory disease that serves as a model for MS. Earlier we and others have shown that, gemfibrozil, a lipid-lowering drug, exhibits therapeutic efficacy in EAE. However, the underlying mechanism was poorly understood. Although gemfibrozil is a known ligand of peroxisome proliferator-activated receptor α (PPARα), here, we established that oral administration of gemfibrozil preserved the integrity of blood–brain barrier (BBB) and blood–spinal cord barrier (BSB), decreased the infiltration of mononuclear cells into the CNS and inhibited the disease process of EAE in both wild type and PPARα^–/–^ mice. On the other hand, oral gemfibrozil was found ineffective in maintaining the integrity of BBB/BSB, suppressing inflammatory infiltration and reducing the disease process of EAE in mice lacking PPARβ (formerly PPARδ), indicating an important role of PPARβ/δ, but not PPARα, in gemfibrozil-mediated preservation of BBB/BSB and protection of EAE. Regulatory T cells (Tregs) play a critical role in the disease process of EAE/MS and we also demonstrated that oral gemfibrozil protected Tregs in WT and PPARα^–/–^ EAE mice, but not PPARβ^–/–^ EAE mice. Taken together, our findings suggest that gemfibrozil, a known ligand of PPARα, preserves the integrity of BBB/BSB, enriches Tregs, and inhibits the disease process of EAE via PPARβ, but not PPARα.

## Introduction

Multiple sclerosis (MS) is the most common human demyelinating disease of CNS. Although the real causes of MS are unknown, data from MS patients and animal models support the idea that it is a myelin-specific CD4^+^ T cell-mediated autoimmune disease. Experimental autoimmune encephalomyelitis (EAE) is commonly employed as a model for MS and as such has been a powerful tool for studying the disease pathogenesis as well as potential therapeutic interventions (Hafler and Weiner, 1989; Martin et al., 1992; Martino and Hartung, 1999; Bruck and Stadelmann, 2003; Pahan, 2010; Robinson et al., 2014; Filippi et al., 2018). Tissue-specific autoimmunity can be inhibited by subpopulations of CD4^+^ cells, termed as regulatory T cells (Tregs). In healthy human beings these cells are capable of suppressing activation and proliferation of self-reactive T cells and thereby inhibition of immune response of self-reactive T cells against self-antigens (Sakaguchi, 2004; Josefowicz et al., 2012; Pahan, 2015). In patients with MS, myelin-reactive T cells overcome the usual restraining mechanism of Tregs, become activated, cross the leaky blood–brain barrier (BBB)/blood–spinal cord barrier (BSB), infiltrate the CNS parenchyma, and initiate a broad-spectrum inflammatory response (Becher et al., 2006; Fletcher et al., 2010; Pahan, 2011).

Gemfibrozil, commonly known as “Lopid” in the pharmacy setting, is well known for its ability to reduce the level of triglycerides in the blood circulation and to decrease the risk of hyperlipidemia (Vahlquist et al., 1995; Buyukcelik et al., 2002). It has been shown that gemfibrozil is capable of suppressing the expression of different proinflammatory molecules in astrocytes and microglia (Pahan et al., 2002; Xu et al., 2005; Jana et al., 2007; Jana and Pahan, 2012). Usually, proinflammatory signaling pathways are kept in check by different anti-inflammatory molecules like SOCS3 and IL-1Ra in many cell types including brain cells (Gabay et al., 2010; Ghosh et al., 2017; Chakrabarti et al., 2021). It has been reported that low-dose gemfibrozil treatment can upregulate the level of both SOCS3 (Ghosh and Pahan, 2012) and IL-1Ra (Corbett et al., 2012) in brain cells. Moreover, gemfibrozil is capable of switching T-helper cells (Dasgupta et al., 2007), altering T cell-to-microglia contact (Roy et al., 2007), and stimulating the expression of myelin-specific genes (Jana et al., 2012). We (Dasgupta et al., 2007), and others (Lovett-Racke et al., 2004) have also demonstrated the protective efficacy of gemfibrozil against EAE in mice. However, the underlying mechanism remained poorly understood.

Although gemfibrozil is an agonist of PPARα, here, by inducing EAE in PPARα^–/–^ and PPARβ^–/–^ mice followed by gemfibrozil treatment, we demonstrated that gemfibrozil treatment required the involvement of PPARβ, but not PPARα, to restore the integrity of BBB and BSB, attenuate the inflammatory infiltration into the spinal cord, upregulate the expression of myelin-specific genes in the spinal cord, and protect mice from EAE. Gemfibrozil also involved PPARβ, but not PPARα, to enrich Foxp3^+^ Tregs in EAE mice. These findings may help in repurposing gemfibrozil for MS.

## Materials and methods

### Reagents

DMEM/F-12, RPMI 1640, Hanks’ balanced salt solution, L-glutamine, 0.05% trypsin, β-mercaptoethanol, and antibiotic/antimycotic were obtained from ThermoFisher (Waltham, MA, USA). Fetal bovine serum or FBS was obtained from Atlas Biologicals (Fort Collins, CO, USA). Gemfibrozil, MOG_35–55_, Solvent Blue 38, cresyl violet acetate, lithium carbonate, Incomplete Freund’s adjuvant (IFA), and all molecular biology–grade chemicals were purchased from Millipore-Sigma (Burlington, MA, USA). Heat-killed *Mycobacterium tuberculosis* (H37RA) was purchased from Difco Laboratories. Alexa-fluor 488 donkey anti-goat, Alexa-fluor 647 donkey anti-rabbit, and Alexa-fluor 488 donkey anti-mouse secondary antibodies used in immunostaining were obtained from Jackson ImmunoResearch (West Grove, PA, USA).

### Induction of EAE

Animal maintenance and experiments were in accordance with National Institute of Health guidelines and were approved by the Institutional Animal Care and Use committee (IACUC# 20-007) of the Rush University of Medical Center, Chicago, IL, United States. EAE was induced by MOG_35–55_ in 8–10-weeks-old male C57/BL6 (WT), PPARα^–/–^, and PPARβ^–/–^ mice via immunization with 100 μg of MOG_35–55_ and 60 μg *M. tuberculosis* in IFA as described (Mondal and Pahan, 2015; Mondal et al., 2017a,^2020^). Mice also received two doses of pertussis toxin (150 ng/mouse) on 0 and 2 dpi (Mondal et al., 2012; Mondal and Pahan, 2015). Animals were observed daily for clinical symptoms as described (Dasgupta et al., 2003, 2004; Brahmachari and Pahan, 2007; Mondal et al., 2009, 2018, 2020) using the following scoring scale: 0, no clinical disease; 0.5, piloerection; 1, tail weakness; 1.5, tail paralysis; 2, hind limb weakness; 3, hind limb paralysis; 3.5, forelimb weakness; 4, forelimb paralysis; and 5, moribund or death.

### Gemfibrozil treatment

Experimental autoimmune encephalomyelitis mice were treated with gemfibrozil at a dose of 7.5 mg/kg/day. Gemfibrozil was mixed in 0.1% methyl cellulose, and EAE mice were gavaged with 100 μl of gemfibrozil-mixed methyl cellulose once daily using a gavage needle (Mondal and Pahan, 2015; Mondal et al., 2017a,^2020^). Therefore, control EAE mice were also gavaged with 100 μl of 0.1% methyl cellulose as vehicle.

### Footprint analysis

**FIGURE 1:**
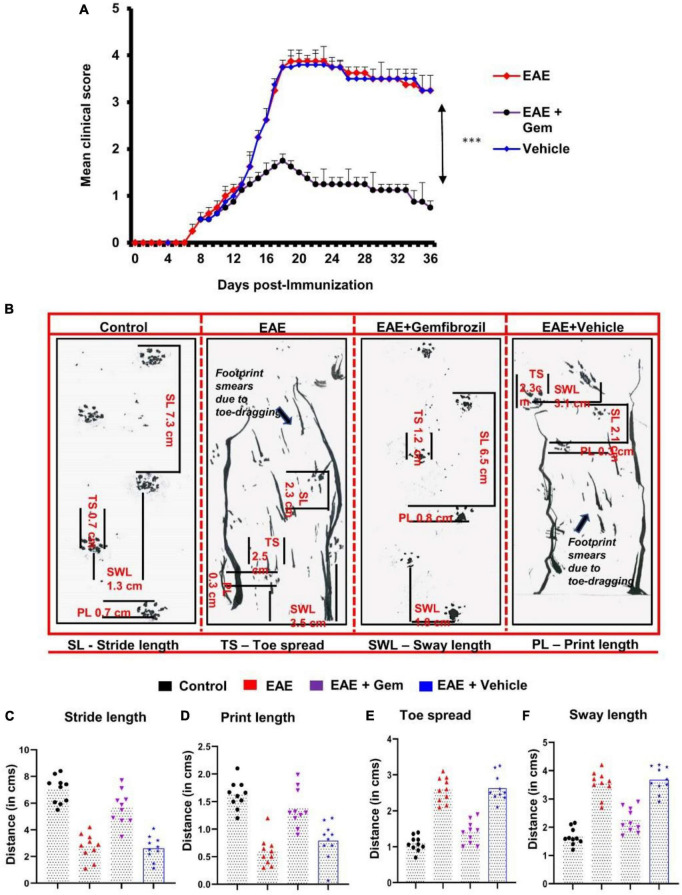
Oral administration of gemfibrozil suppresses clinical symptoms of EAE and attenuates motor behavioral impairment in chronic EAE mice. EAE was induced in 8–10-weeks-old male C57/BL6 mice by MOG_35–55_ immunization, and from 8 dpi, mice were treated with gemfibrozil (7.5 mg/kg/day) orally via gavage. Mice were scored daily until 36 dpi **(A)**. Motor coordination of experiment animals was evaluated by gait analysis and footprints of animals on the gangway are shown **(B)**. Stride length **(C)**, print length **(D)**, toe spread **(E)**, and sway length **(F)** of mice obtained from the gait analysis are calculated manually. Data are represented as mean ± SEM of five mice per group. One-way ANOVA followed by Tukey’s multiple comparison test was performed for statistical analyses. ***p < 0.001.

Gait analysis using footprint measurement is an important behavioral test used to study the motor function deficits in different neurological disease models. We conducted footprint analysis to examine the recovery of motor functions. The details of methodology of footprint technique for mice have been published in our earlier studies (Rangasamy et al., 2021). The footprint recordings of mice were conducted using non-toxic black ink and white paper. Briefly, the fore and hind paws of mice were gently dipped in non-toxic black ink and paw prints were recorded while they walk on flat board of wide runway covered with white paper. The paw prints and measurement of different gait variables are illustrated in Figure 1B. Three to four trials for each mouse were performed to obtain consistent prints and measured for (1) stride length (the distance between the paw print to subsequent print of the same foot either right or left side); (2) toe-spread (the distance between the first toe to fifth toe); (3) print length (the length of forelimb or hindlimb individually); and (4) sway length (the distance between individual left and right hindlimb prints obtained by measuring the perpendicular area spread apart). We measured the paw prints in centimeters and at least 15 steps were recorded for each mouse. Mean value was used for statistical analysis.

### Isolation of splenocytes

Spleens isolated from normal or EAE mice were placed into a cell strainer and mashed with a syringe plunger as described (Mondal et al., 2020). Resulting single-cell suspensions were treated with RBC lysis buffer (Sigma-Aldrich), washed, and cultured in 12-well plates in RPMI 1640 supplemented with 10% FBS, 50 μM 2-ME, 2 mM L-glutamine, 100 U/ml penicillin, and 100 μg/ml streptomycin. In addition to these, splenocytes isolated from either MOG-immunized mice or EAE mice also received 50 μg/ml MOG.

### Histological analysis

Histological analysis was performed in spinal cord sections of EAE mice as we described previously (Mondal et al., 2009, 2018; Mondal and Pahan, 2015). At the peak of the acute phase, mice were anesthetized and perfused with PBS (pH 7.4) and then with 4% (w/v) paraformaldehyde solution in PBS followed by dissection of whole spinal cord and brain from each mouse. The tissues were further fixed and then divided into two halves: one-half was used for histology analysis whereas the other half for myelin staining as described earlier (Brahmachari and Pahan, 2007; Mondal et al., 2009, 2018; Roy and Pahan, 2015). For histological analysis, routine histology was performed to obtain perivascular cuffing and morphological details of spinal cord. Paraformaldehyde-fixed tissues were embedded in paraffin, and serial sections (4 μm) cut. Sections were stained with conventional hematoxylin and eosin (H&E) staining method. Digital images were collected under bright field setting using a ×40 objective. Slides were assessed in a blinded fashion for inflammation by three examiners in different anatomical compartments (meninges and parenchyma). Inflammation was scored using the following scale as described: for meninges and parenchyma: 0, no infiltrating cells; 1, few infiltrating cells; 2, numerous infiltrating cells; and 3, widespread infiltration. For vessels: 0, no cuffed vessel; 1, one or two cuffed vessels per section; 2, three to five cuffed vessels per section; and 3, more than five cuffed vessels per section. At least six serial sections of each spinal cord and cerebellar tissues from each of five mice per group were scored.

### Assessment of blood–brain barrier and blood–spinal cord barrier permeability

It was performed as described before (Mondal et al., 2009, 2017b,^2018^; Mondal and Pahan, 2015). Briefly, on 20 dpi (acute phase), mice received 200 μl of 20 μM Alexa 680-SE-NIR dye (Invitrogen) via tail vain. After 2 h, mice were scanned in Odyssey (ODY-0854, Licor, Inc.) infrared scanner at 700- and 800-nm channels followed by perfusion with 4% paraformaldehyde. The spinal cord and different regions of brain were scanned in Odyssey infrared scanner. The red background came from an 800 nm filter, whereas the green signal was from Alexa 680 dye at 700 nm channel. The density of the Alexa 680 signal was quantified with the help of Quantity One, version 4.6.2 software using the volume contour tool analysis module.

### Flow cytometry

Single-cell suspensions isolated from mouse spleen were stained with Zombie Aqua™ Fixable Viability Kit (Bioligand) according to the manufacturer’s instructions as described before (Mondal et al., 2018, 2020). Cells were washed with FACS buffer (ThermoFisher) and stained with CD3-Brilliant Violet 605, CD4-FITC, and CD8-APC-Cy7 (Bioligand) for extracellular stains. For intracellular staining, cells were stained according to manufacturer’s instructions using the eBioscience™ Foxp3/Transcription Factor Staining Buffer set (ThermoFisher). Cells were then stained with anti-Foxp3-APC (ThermoFisher). Multicolor flow cytometric analyses were performed using the LSRFortessa analyzer (BD Biosciences) and analyzed using the FlowJo Software (v10).

### Statistical analysis

Analyses were performed by GraphPad Prism 7.02 software. ANOVA were used to compare different groups. Wherever required, repeated measures one-way ANOVA was employed. Data are shown as means ± SEM.

## Results

### Oral administration of gemfibrozil suppresses clinical symptoms, disease severity, and improves locomotor activity of EAE mice

We have reported that gemfibrozil reduces clinical symptoms and disease severity of relapsing-remitting EAE in female SJL/J mice (Dasgupta et al., 2007). Here, we examined whether oral gemfibrozil could inhibit the clinical symptoms and disease severity in chronic model of EAE in mice. EAE was induced in 8–10-weeks-old male C57/BL6 mice by MOG_35–55_ immunization and from 8 day post-immunization (dpi), mice were treated with gemfibrozil daily by oral gavage. Similar to that found in RR-EAE mice, gemfibrozil treatment inhibited the clinical symptoms of chronic EAE in mice (Figure 1A). Although significant inhibition of clinical symptoms was observed within 8 days of gemfibrozil treatment, greater inhibition was seen in further days of treatment, which was maintained throughout the duration of experiment (Figure 1A).

Measurement of footprints using different variables plays a vital role in the assessment of motor function deficits. We performed the footprint analysis in mice on 20 dpi and compared the gait functions of untreated EAE mice with that of gemfibrozil treated EAE mice. We observed significant changes in different footprint variables from the recorded paw prints of untreated EAE mice when compared with that of gemfibrozil treated EAE mice (Figures 1B–F). Stride length delineates the capacity of the forelimb or hindlimb to support the animal’s weight while performing the locomotion. As expected, we found a significant decrease in the stride length of EAE mice when compared to control mice. However, EAE mice treated with gemfibrozil, but not the vehicle, improved gait functions and increased the stride length near to control animals (Figures 1B, C). Additionally, we also frequently noticed dragging of toes in both forepaw and hind paw of EAE mice while recording the footprints, indirectly denoting that these mice exhibit severe deficits in locomotion (arrow indicates Figure 1B). However, oral administration of gemfibrozil significantly reduced the dragging of toes in both limbs of EAE mice and improved the motor function deficits.

Print length provides evidence of how firmly the foot is placed on a flat surface. As evident from Figures 1B, D, EAE mice exhibited significant decrease in print length as compared to control mice. Such a decrease in print length measurement denotes the inability of EAE mice to place the complete foot on the flat surface. However, following the gemfibrozil treatment, EAE mice exhibited a significant increase in measurement of print length to recover close to that of control animals (Figures 1B, D). Increase in toe spread and sway length measurement indicates that EAE mice exhibit poor performance in maintaining their body balance while walking on the flat board. We noticed a significant increase in toe spread (Figures 1B, E) and sway length (Figures 1B, F) of EAE mice as compared to control animals. However, EAE mice treated with gemfibrozil demonstrated significant increase in toe spread and sway length measurement and improved the capability to balance its body weight while performing locomotion (Figures 1B, E, F).

### Gemfibrozil requires PPARβ, but not PPARα, to suppress EAE

**FIGURE 2:**
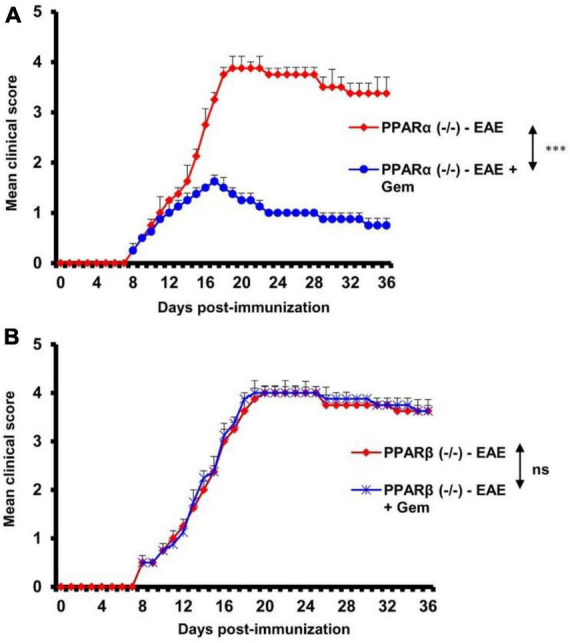
Gemfibrozil protects mice from EAE via PPARβ, but not PPARα. EAE was induced in 8–10-weeks-old male PPARα^–/–^ **(A)** and PPARβ^–/–^ **(B)** mice (*n* = 5 per group) by MOG immunization followed by treatment with gemfibrozil (7.5 mg/kg/day) orally via gavage starting from 8 dpi. Clinical scores were recorded until 36 dpi. Data are represented as mean ± SEM of five mice per group. One-way ANOVA followed by Tukey’s multiple comparison test was performed for statistical analyses. ***p < 0.001.

Next, to investigate the mechanism by which gemfibrozil reduced EAE symptoms in mice, we investigated the role of PPARs, nuclear hormone receptors that are known to be involved in various immunomodulatory mechanisms (Dunn et al., 2010; Georgiadi and Kersten, 2012; Alex et al., 2013). Since gemfibrozil is a known agonist of PPARα, to delineate its role in gemfibrozil-mediated protection of EAE, we induced EAE in PPARα^–/–^ mice by MOG immunization followed by gemfibrozil treatment. For comparison, we also used PPARβ^–/–^ mice. As expected, we observed clinical symptoms of EAE in both PPARα^–/–^ (Figure 2A) and PPARβ^–/–^ (Figure 2B) mice upon MOG immunization. However, oral gemfibrozil treatment via gavage starting from 8 dpi reduced clinical symptoms of EAE in PPARα^–/–^ mice (Figure 2A), specifying that PPARα is not involved in gemfibrozil-mediated suppression of EAE symptoms. On the other hand, clinical symptoms of EAE were not inhibited by gemfibrozil treatment in PPARβ^–/–^ mice (Figure 2B), indicating that gemfibrozil requires PPARβ to suppress EAE symptoms.

### Oral gemfibrozil preserves the integrity of the blood–brain barrier and blood–spinal cord barrier in mice with chronic EAE via PPARβ, but not PPARα

The BBB and BSB are a tightly packed layer of cells that line the blood vessels in the brain and spinal cord (Abbott et al., 2006; Sweeney et al., 2018). Functions of these important barriers are to prevent the entry of large molecules, immune cells, and disease-causing organisms into the CNS, while allowing some essential molecules to the CNS microenvironment (Abbott et al., 2006; Sweeney et al., 2018). However, it has been reported that the integrity of both BBB and BSB is compromised during active MS and EAE (Mondal et al., 2018, 2020; Sweeney et al., 2018; Faissner et al., 2019). Therefore, we investigated whether gemfibrozil treatment modulated the integrity of BBB and BSB. We injected a near-infrared dye (Alexa Fluor 680-SE-NIR) via tail-vein and 2 h after the injection, live mice were scanned in an Odyssey infra-red scanner (ODY-0854, Licor, Inc.) at 700- and 800-nm channels.

**FIGURE 3:**
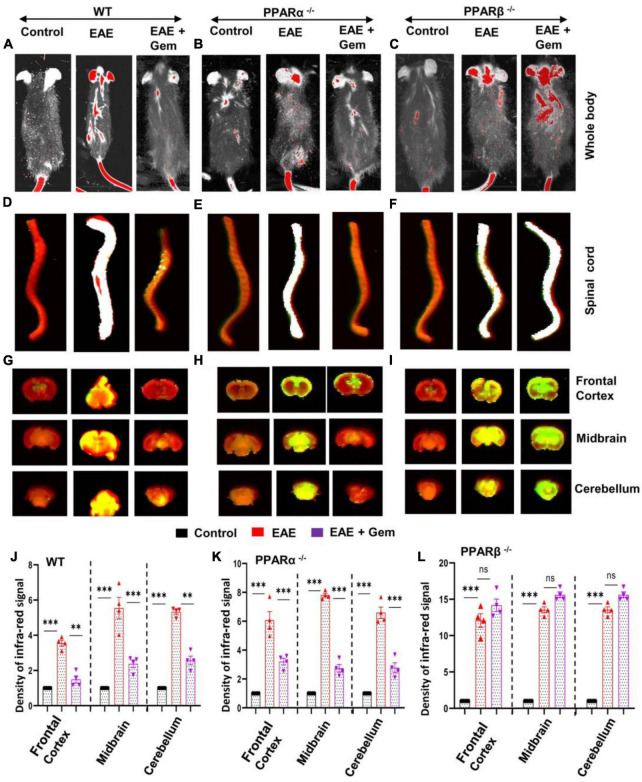
Oral gemfibrozil requires PPARβ, but not PPARα, to restore the integrity of blood–brain barrier (BBB) and blood–spinal cord barrier (BSB) in EAE mice. EAE was induced in 8–10-weeks-old male WT **(A,D,G,J)**, PPARα^–/–^ **(B,E,H,K)**, and PPARβ^–/–^ **(C,F,I,L)** mice (*n* = 4 per group) by MOG immunization followed by treatment with gemfibrozil (7.5 mg/kg/day) orally via gavage starting from 8 dpi. On 20 dpi (acute phase), mice received 200 μl of 20 μM Alexa Fluor 680-SE-NIR dye (Invitrogen) via the tail vain. After 2 h, mice were scanned in an Odyssey (ODY-0854; LICOR Biosciences) infrared scanner at the 700- and 800-nm channels **(A–C)**. Mice were perfused with 4% paraformaldehyde. Spinal cord **(D–F)**, and frontal cortex, midbrain, and cerebellum **(G–I)** were scanned in an Odyssey infrared scanner. The red background came from an 800-nm filter, whereas the green signal was from Alexa Fluor 680 dye at the 700-nm channel. The density of Alexa Fluor 680 signal in different parts of the brain (**J**, WT; **K**, PPARα^–/–^; and **L**, PPARβ^–/–^) was quantified with the help of Quantity One, version 4.6.2 software, using the volume contour tool analysis module. Data are expressed as the mean ± SEM of four mice per group. One-way ANOVA followed by Tukey’s multiple comparison test was performed for statistical analyses. **p < 0.01; ***p < 0.001; ns, not significant.

We observed that infra-red signals were not visible on areas over the brain and the spinal cord in control HBSS-injected mice (Figure 3A). On the other hand, in EAE mice, infra-red signals were detected on areas over the brain and the spinal cord (Figure 3A), suggesting possible breakdown of BBB and BSB in EAE mice. However, gemfibrozil treatment strongly inhibited the entry of infra-red dye into the CNS of EAE mice (Figure 3A). To confirm these results further, the spinal cord and different parts of the brain (frontal cortex, midbrain, and cerebellum) were scanned for infra-red signals in an Odyssey infra-red scanner. Consistent to live mice results, no infra-red signal was noticed in the spinal cord (Figure 3D), frontal cortex, midbrain, and cerebellum (Figures 3G, J) of control HBSS-treated mice, but significant amount of infra-red dye was visible in CNS tissues of EAE mice (Figures 3D, G, J). Again, treatment of EAE mice with gemfibrozil led to marked attenuation of the entry of infra-red dye into the spinal cord and different parts of the brain (Figures 3D, G, J). These results suggest that gemfibrozil treatment prevents the integrity of BBB and BSB in EAE mice.

Next, we investigated mechanisms by which gemfibrozil restored the integrity of BBB and BSB in EAE mice. Since gemfibrozil required PPARβ, but not PPARα, to reduce clinical symptoms of EAE, we also investigated the effect of gemfibrozil on the status of BBB and BSB in PPARα^–/–^ and PPARβ^–/–^ EAE mice. While gemfibrozil treatment led to strong decrease in the entry of infra-red dye into the spinal cord, frontal cortex, midbrain, and cerebellum of PPARα^–/–^ EAE mice (Figures 3B, E, H, K), it remained unable to inhibit the entry of infra-red dye into the spinal cord and different parts of the brain of PPARβ^–/–^ EAE mice (Figures 3C, F, I, L), indicating that gemfibrozil requires PPARβ, but not PPARα, to maintain the integrity of BBB and BSB in EAE mice.

### Oral gemfibrozil inhibits inflammatory infiltration into the CNS of EAE mice via PPARβ, but not PPARα

**FIGURE 4:**
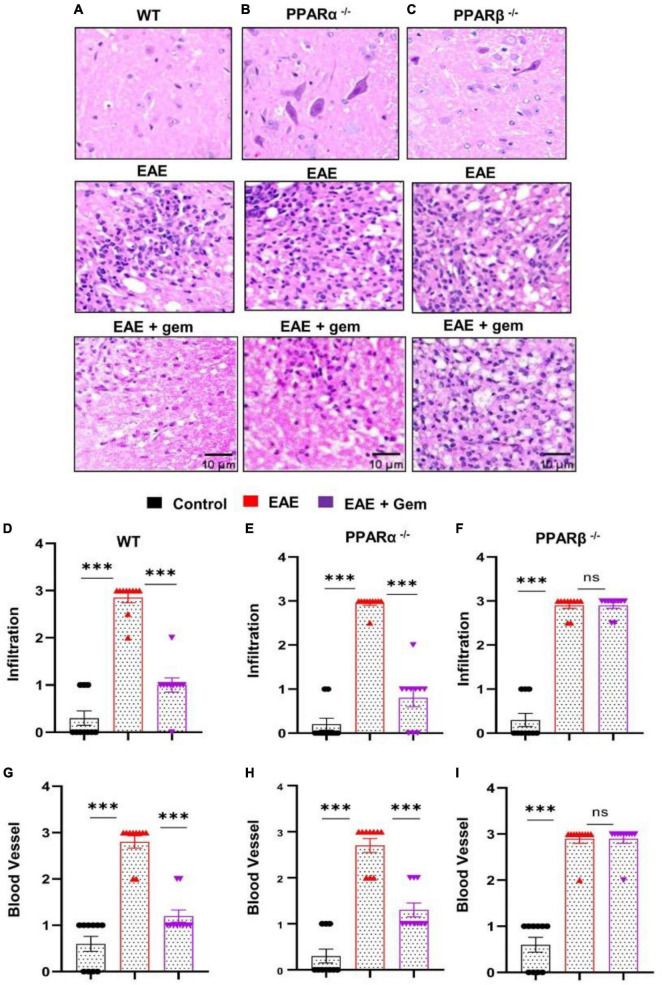
Oral gemfibrozil requires PPARβ, but not PPARα, to suppress inflammatory infiltration into the spinal cord of EAE mice. EAE was induced in 8–10-weeks-old male WT **(A,D,G)**, PPARα^–/–^ **(B,E,H)**, and PPARβ^–/–^ **(C,F,I)** mice (*n* = 5 per group) by MOG immunization followed by treatment with gemfibrozil (7.5 mg/kg/day) orally via gavage starting from 8 dpi. On 20 dpi (acute phase), spinal cord sections were stained with H&E and digital images were collected under bright field setting **(A–C)**. Infiltration **(D–F)** and cuffed vessel **(G–I)** were represented quantitatively by using a scale as described in section “Materials and methods.” Data are expressed as the mean ± SEM of five mice per group. One-way ANOVA followed by Tukey’s multiple comparison test was performed for statistical analyses. ***p < 0.001; ns, not significant.

It is believed that MS and EAE are caused by the infiltration of autoreactive T cells and associated mononuclear cells, like macrophages, into the CNS (Steinman, 1996; Benveniste, 1997; Pahan, 2010, 2011). We examined whether gemfibrozil treatment could reduce the infiltration of inflammatory cells into the spinal cord of EAE mice. Spinal cord sections of WT control, EAE (20 dpi), and gemfibrozil-treated EAE mice receiving gemfibrozil from 8 dpi, were stained with H&E. As expected, we observed widespread infiltration of inflammatory cells into the spinal cord of EAE mice (Figure 4A). On the other hand, gemfibrozil treatment markedly inhibited the infiltration of inflammatory cells into the spinal cord of WT EAE mice (Figure 4A). Blinded quantitation of spinal cord histology also showed that gemfibrozil could reduce immune cell infiltration (Figure 4D) and the appearance of cuffed vessels (Figure 4G) in spinal cord of WT EAE mice.

Next, we examined mechanisms by which gemfibrozil treatment could inhibit inflammatory infiltration into the spinal cord of EAE mice. Since gemfibrozil involved PPARβ, but not PPARα, to inhibit the breakdown of BBB and BSB, we performed H&E staining in spinal cord sections of gemfibrozil-treated and untreated PPARα^–/–^ and PPARβ^–/–^ EAE mice. Similar to the restoration of integrities of BBB and BSB, gemfibrozil inhibited the immune cell infiltration (Figures 4B, E) and the appearance of cuffed vessels (Figure 4H) in the spinal cord of PPARα^–/–^ EAE mice. However, gemfibrozil treatment had no effect on inflammatory infiltration (Figures 4C, F) and the appearance of cuffed vessels (Figure 4I) in the spinal cord of PPARβ^–/–^ EAE mice. These results clearly suggest that gemfibrozil requires PPARβ, but not PPARα, to suppress inflammatory infiltration into the spinal cord of EAE mice.

### Oral gemfibrozil upregulates myelin-specific genes in the CNS of EAE mice via PPARβ, but not PPARα

**FIGURE 5:**
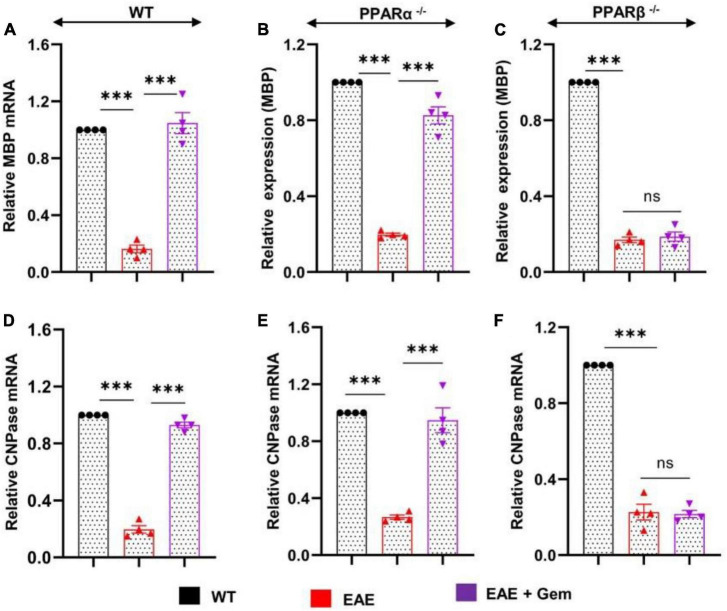
Oral gemfibrozil upregulates the expression of myelin-specific genes in the spinal cord of EAE mice via PPARβ, but not PPARα. EAE was induced in 8–10-weeks-old male WT **(A,D)**, PPARα^–/–^ **(B,E)**, and PPARβ^–/–^ **(C,F)** mice (*n* = 4 per group) by MOG immunization followed by treatment with gemfibrozil (7.5 mg/kg/day) orally via gavage starting from 8 dpi. On 20 dpi (acute phase), the mRNA expression of MBP **(A–C)** and CNPase **(D–F)** was monitored by real-time PCR. Data are expressed as the mean ± SEM of four mice per group. One-way ANOVA followed by Tukey’s multiple comparison test was performed for statistical analyses. ***p < 0.001; ns, not significant.

Multiple sclerosis is a demyelinating disorder, and it is believed that the infiltration of blood mononuclear cells and associated neuroinflammation may promote CNS demyelination (Steinman, 1996; Benveniste, 1997). Several myelin-specific molecules like myelin basic protein (MBP), 2′,3′-cyclic nucleotide 3′-phosphodiesterase (CNPase), myelin oligodendrocyte glycoprotein (MOG), and proteolipid protein (PLP) serve as markers of myelin (Jana and Pahan, 2005, 2013). As expected, we found a significant decrease in mRNA expression of MBP (Figure 5A) and CNPase (Figure 5D) in the spinal cord of WT EAE mice as compared to WT control mice. However, gemfibrozil treatment markedly restored the mRNA expression of MBP (Figure 5A) and CNPase (Figure 5D) in the spinal cord of WT EAE mice. When we tested the recovery of myelin-specific genes in the spinal cord of PPARα^–/–^ EAE mice following gemfibrozil treatment, we found that gemfibrozil was able to upregulate the mRNA expression of MBP (Figure 5B) and CNPase (Figure 5E) in the spinal cord of PPARα^–/–^ EAE mice. On the other hand, gemfibrozil treatment could not upregulate the mRNA expression of MBP (Figure 5C) and CNPase (Figure 5F) in the spinal cord of PPARβ^–/–^ EAE mice. These results suggest that gemfibrozil upregulates the expression of myelin-specific genes in the spinal cord of EAE mice via PPARβ, but not PPARα.

### Gemfibrozil protects regulatory T cells in EAE mice via PPARβ, but not PPARα

**FIGURE 6:**
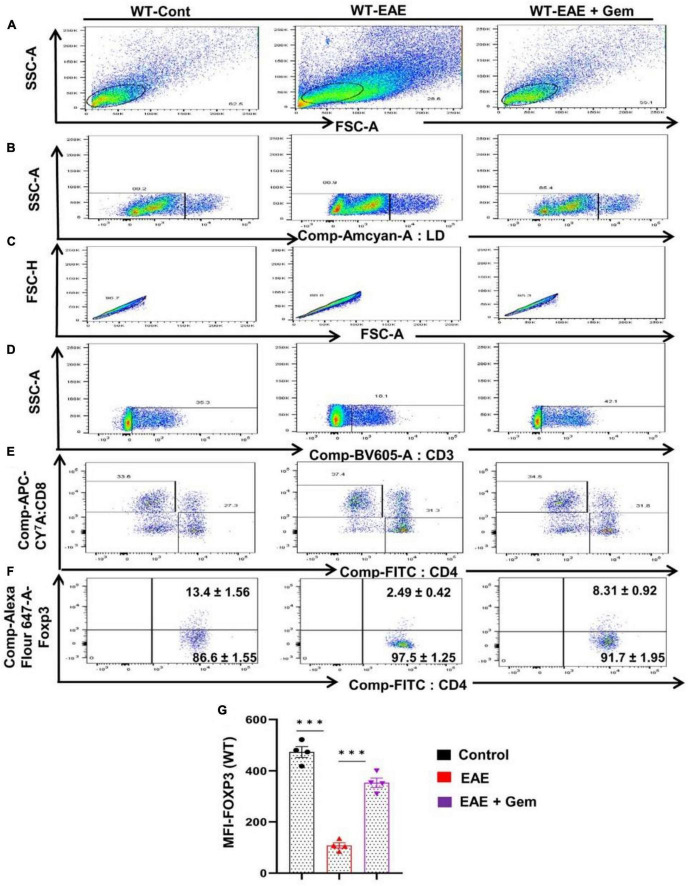
Oral administration of gemfibrozil enriches the regulatory T cells in chronic EAE mice. Splenocytes isolated from control, EAE (20 dpi), and gemfibrozil treated EAE mice (receiving gemfibrozil from 8 dpi) were stained with Zombie Aqua™ with Zombie Aqua™ Fixable Viability Kit (Biolegend). Cells were washed with FACS buffer (ThermoFisher) and stained with CD3-BV, CD4-FITC, and CD8 APC Cy7 (Biolegend) for extra cellular stains. The eBioscience™Foxp3/Transcription Factor Staining Buffer set (ThermoFisher) was used for intracellular staining of Foxp3-APC. Multicolor flow cytometric analyses were performed using LSRFortessa analyzer (BD Biosciences) and analyzed using FlowJo Software (v10). SSC-A versus FSC-A **(A)**, dead cell exclusion **(B)**, selection of singlets from the FSC-H versus FSC-A dot plot **(C)**, divided into T cells based on surface expression of CD3 **(D)**, the dot plot on the lower right and upper left show a clear demarcation of CD4 and CD8 cell populations **(E)**, and analysis of Foxp3^+^ cells from CD4^+^ cells **(F)**. MFI of Foxp3 in CD4^+^ cells **(G)**. Data are expressed as the mean ± SEM of four mice per group. One-way ANOVA followed by Tukey’s multiple comparison test was performed for statistical analyses. ***p < 0.001.

Multiple sclerosis is an autoimmune disorder and an immune dysregulation led by autoimmune T cells is believed to play an important role in this disease (Steinman, 1996; Benveniste, 1997; Brahmachari and Pahan, 2008). Fortunately, we have been also endowed with Tregs, an inhibitory T lymphocyte subset characterized by the transcription factor Foxp3, to keep autoimmune T cells in check (Walsh et al., 2004). It has been reported that there is a reduction of Tregs in patients with MS and other autoimmune disorders (Venken et al., 2010), ultimately allowing over-activation of autoimmune T cells. Therefore, upregulation of Tregs might be useful for MS/EAE and we examined the status of Tregs *in vivo* in EAE mice on 20 dpi receiving gemfibrozil from 8 dpi. As evident from a FACS dot plot (Figures 6A–F) and mean fluorescence intensity (MFI) (Figure 6G), there was a significant reduction in the CD4^+^ Foxp3^+^ population of T cells in EAE splenocytes as compared to splenocytes isolated from normal mice. However, upregulation of CD4^+^ Foxp3^+^ population of T cells was found in EAE mice by gemfibrozil treatment (Figures 6A–G).

**FIGURE 7:**
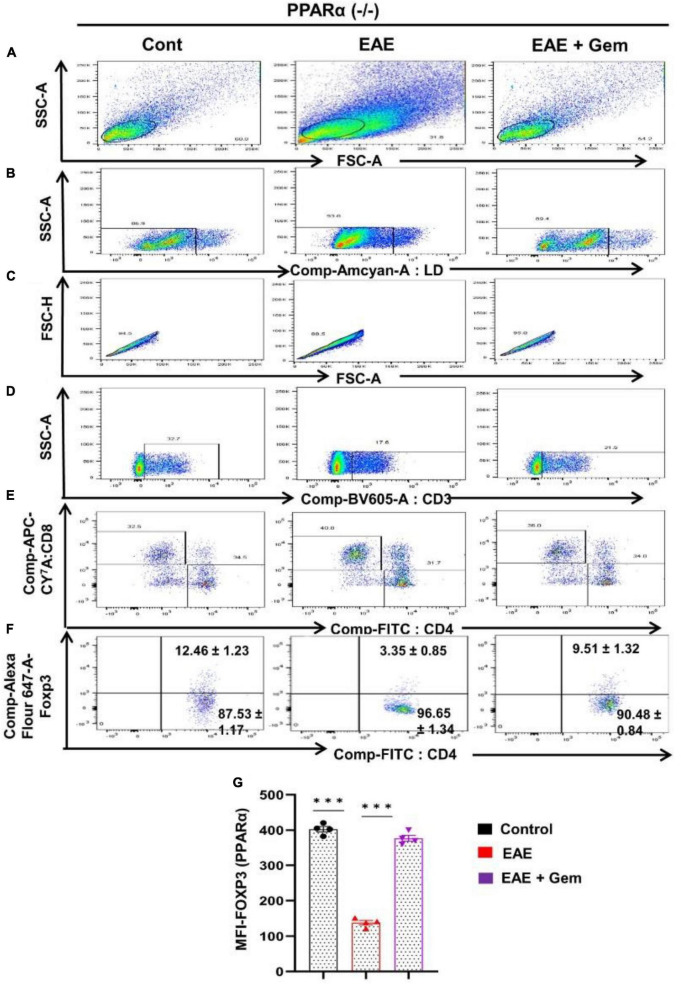
Gemfibrozil treatment enriches Tregs in PPARα^–/–^ EAE mice. Splenocytes isolated from PPARα^–/–^ control, PPARα^–/–^ EAE (20 dpi), and gemfibrozil treated PPARα^–/–^ EAE mice (receiving gemfibrozil from 8 dpi) were stained with Zombie Aqua™ with Zombie Aqua™ Fixable Viability Kit (Biolegend). Cells were washed with FACS buffer (ThermoFisher) and stained with CD3-BV, CD4-FITC, and CD8 APC Cy7 (Biolegend) for extra cellular stains. The eBioscience™Foxp3/Transcription Factor Staining Buffer set (ThermoFisher) was used for intracellular staining of Foxp3-APC. Multicolor flow cytometric analyses were performed using LSRFortessa analyzer (BD Biosciences) and analyzed using FlowJo Software (v10). SSC-A versus FSC-A **(A)**, dead cell exclusion **(B)**, selection of singlets from the FSC-H versus FSC-A dot plot **(C)**, divided into T cells on the basis of surface expression of CD3 **(D)**, the dot plot on the lower right and upper left show a clear demarcation of CD4 and CD8 cell populations **(E)**, and analysis of Foxp3^+^ cells from CD4^+^ cells **(F)**. MFI of Foxp3 in CD4^+^ cells **(G)**. Data are expressed as the mean ± SEM of four mice per group. One-way ANOVA followed by Tukey’s multiple comparison test was performed for statistical analyses. ***p < 0.001.

**FIGURE 8:**
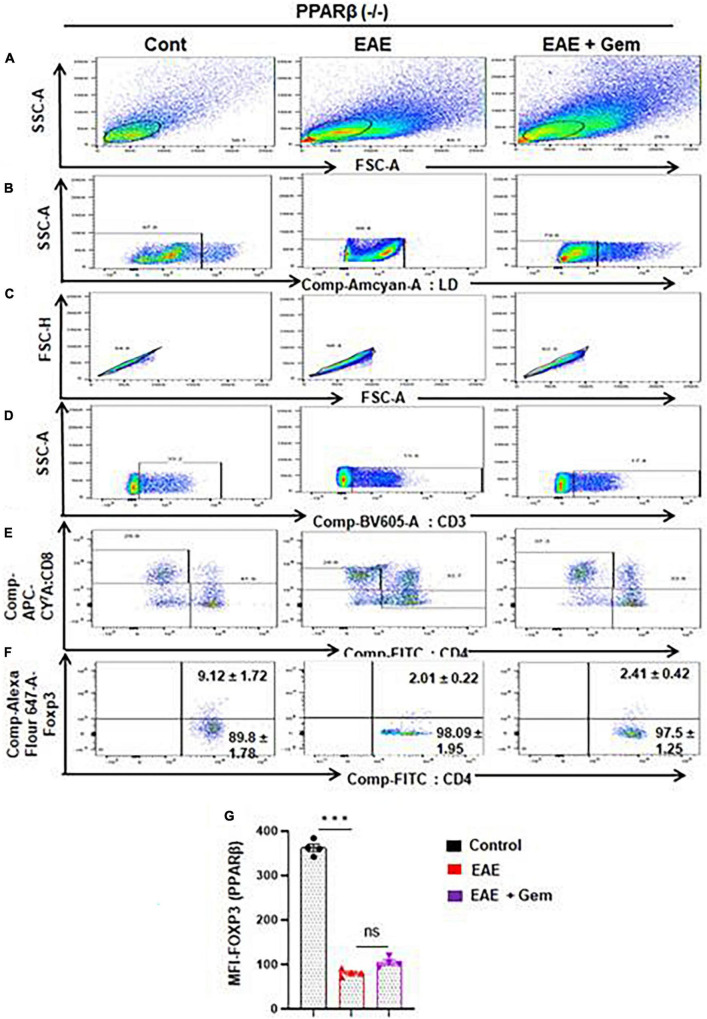
Gemfibrozil treatment does not upregulate Tregs in PPARβ^–/–^ EAE mice. Splenocytes isolated from PPARβ^–/–^ control, PPARβ^–/–^ EAE (20 dpi), and gemfibrozil treated PPARβ^–/–^ EAE mice (receiving gemfibrozil from 8 dpi) were stained with Zombie Aqua™ with Zombie Aqua™ Fixable Viability Kit (Biolegend). Cells were washed with FACS buffer (ThermoFisher) and stained with CD3-BV, CD4-FITC, and CD8 APC Cy7 (Biolegend) for extra cellular stains. The eBioscience™Foxp3/Transcription Factor Staining Buffer set (ThermoFisher) was used for intracellular staining of Foxp3-APC. Multicolor flow cytometric analyses were performed using LSRFortessa analyzer (BD Biosciences) and analyzed using FlowJo Software (v10). SSC-A versus FSC-A **(A)**, dead cell exclusion **(B)**, selection of singlets from the FSC-H versus FSC-A dot plot **(C)**, divided into T cells based on surface expression of CD3 **(D)**, the dot plot on the lower right and upper left show a clear demarcation of CD4 and CD8 cell populations **(E)**, and analysis of Foxp3^+^ cells from CD4^+^ cells **(F)**. MFI of Foxp3^+^ in CD4^+^ cells **(G)**. Data are expressed as the mean ± SEM of four mice per group. One-way ANOVA followed by Tukey’s multiple comparison test was performed for statistical analyses. ***p < 0.001; ns, not significant.

Next, we next examined whether gemfibrozil required PPARα and/or PPARβ for this important function. Therefore, EAE was induced in PPARα^–/–^ and PPARβ^–/–^ mice, and gemfibrozil treatment began at 8 dpi for 10 days followed by analysis of Tregs responses in the spleen. Similar to the enrichment of Tregs in WT-EAE mice, gemfibrozil treatment increased the Foxp3^+^ CD4^+^ population of T cells in spleen of PPARα^–/–^ EAE mice as evident from FACS dot plots (Figures 7A–F) and MFI (Figure 7G). In contrast, gemfibrozil treatment could not upregulate the Foxp3^+^ CD4^+^ population of T cells in the spleen of PPARβ^–/–^ EAE mice (Figures 8A–F). MFI calculations also supported this finding (Figure 8G). These results clearly suggest that gemfibrozil requires PPARβ, but not PPARα, for the upregulation of Tregs in EAE mice.

## Discussion

Multiple sclerosis is an autoimmune disorder of the CNS in which myelin components are particularly targeted by the immune system, resulting in demyelination of axons and associated debilitating symptoms that vary over time. There is no cure for MS, but treatments are available which typically focus on reducing the frequency and severity of MS episodes. The first biologic agent used in the treatment of MS was IFN-β, followed by glatiramer-acetate, monoclonal antibodies, fingolimod, and others (Rommer et al., 2019; Li et al., 2020). Each of these has a number of side effects (Rommer et al., 2019; Li et al., 2020). Therefore, describing therapies for MS that are effective and non-toxic is an important area of research. Gemfibrozil known as “Lopid” in the pharmacy is an FDA-approved lipid-lowering drug that has been well tolerated in human and animal studies. It has been shown that gemfibrozil can ameliorate the disease process of EAE in mice. However, underlying mechanisms remained unclear. Establishing molecular mechanisms for anti-MS activities of gemfibrozil may help in repurposing this drug for MS.

Peroxisome proliferator-activated receptors (PPARs) are members of a nuclear hormone receptor superfamily that include receptors for steroids, retinoids, and thyroid hormone, all of which are known to affect the immune response (Rommer et al., 2019; Li et al., 2020). Three PPAR subtypes exist (α, β, and γ) and they exhibit different tissue distribution as well as different ligand specificities (Rommer et al., 2019; Li et al., 2020). The functions of PPARα and PPARγ have been extensively documented because these isoforms are activated by molecules clinically used as hypolipidemic and antidiabetic compounds, respectively. While PPARα is involved in the regulation of lipid metabolism, it is well known that adipose tissue plays a crucial role in antidiabetic action of PPARγ. In addition, PPARγ can directly affect liver and pancreatic β-cells to improve glucose homeostasis (Marcus et al., 1993; Roy and Pahan, 2015; Wagner and Wagner, 2020b). On the other hand, PPARβ governs a variety of biological processes including fatty acid synthesis, oxidative metabolism in white adipose tissue, etc., thus controlling the development of several chronic diseases, including diabetes, obesity, and atherosclerosis (Mirza et al., 2019; Wagner and Wagner, 2020a; Qiu et al., 2023).

Although gemfibrozil is a known ligand of PPARα, here, we have established the involvement of PPARβ, but not PPARα, in gemfibrozil-mediated protection of mice from EAE. The BBB/BSB is a multilayered complex barrier, disruption of the BBB/BSB is frequently observed during the first years after MS onset when the disease is characterized by MS relapses and gadolinium enhancing lesions (Waubant, 2006; Rovira et al., 2023). Therefore, one of the criteria to classify MS as an active one at the onset of the disease for the selection of the appropriate therapy is BBB breakdown, reflecting the degree of MS aggressiveness (Rovira et al., 2023). Although the mechanisms leading to the leakiness of BBB/BSB are poorly known, the entry of infra-red dye into the frontal cortex, midbrain, cerebellum, and spinal cord of WT and PPARα^–/–^, but not PPARβ^–/–^, EAE mice by gemfibrozil suggest the involvement of PPARβ, but not PPARα, for maintaining the integrity of BBB and BSB in EAE mice. Breakdown of BBB/BSB often allows blood mononuclear cells to enter the CNS, leading to neuroinflammation and demyelination. Consistent to PPARβ-dependent inhibition of BBB/BSB leakage, gemfibrozil reduced inflammatory infiltration and increased the expression of myelin-specific genes in the CNS of EAE mice through the involvement of PPARβ, but not PPARα. Therefore, as it appears that the activation of PPARβ may be an important pathway for restoring the integrity of BBB/BSB, inhibiting the infiltration of inflammatory cells into the CNS and inhibiting demyelination.

Regulatory T cells, an inhibitory T lymphocyte subset characterized by the transcription factor Foxp3, plays a critical role in modulating the immune response to self and foreign antigens and helping to prevent autoimmune disease like MS and EAE (Viglietta et al., 2004; Huan et al., 2005; Brahmachari and Pahan, 2009). CD4^+^ CD25^+^ Foxp3^+^ T cells are considered as the major common phenotype of Tregs. Tregs are sufficient to reduce the activity of autoreactive T cells. During autoimmune pathogenesis, the immune system is dysregulated, resulting in a significant loss in the activity and number of Tregs and leading to proliferation of autoreactive T cells and subsequent autoimmune attack (Viglietta et al., 2004; Huan et al., 2005). Therefore, dysfunction of Tregs could be a major cause for the activation of myelin-reactive T cells in MS. The molecular mechanism by which Tregs are suppressed in MS and other autoimmune diseases is poorly understood. Although down regulation of Tregs during an autoimmune disease is poorly understood. Our data suggested that gemfibrozil treatment markedly inhibited the loss of Foxp3 expression in WT and PPARα^–/–^, but not PPARβ^–/–^, EAE mice. Therefore, gemfibrozil treatment protects the loss of Tregs in EAE mice via PPARβ, but not PPARα.

## Conclusion

In summary, we have demonstrated that oral administration of gemfibrozil attenuates the disease process of chronic EAE, preserves the integrity of BBB/BSB, inhibits the infiltration of mononuclear cells, and upregulates the expression of myelin-specific genes in WT and PPARα^–/–^, but not PPARα^–/–^, EAE mice. These results highlight a novel immunomodulatory role of gemfibrozil and PPARβ that may be explored for therapeutic intervention in MS.

## Data availability statement

The raw data supporting the conclusions of this article will be made available by the authors, without undue reservation.

## Ethics statement

The animal study was approved by the Institutional Animal Care and Use committee of the Rush University of Medical Center, Chicago, IL, United States. The study was conducted in accordance with the local legislation and institutional requirements.

## Author contributions

SM: Data curation, Formal analysis, Investigation, Methodology, Visualization, Writing – original draft. MS: Data curation, Formal analysis, Investigation, Validation, Visualization, Writing – original draft. SR: Data curation, Formal analysis, Investigation, Validation, Writing – original draft. KP: Conceptualization, Funding acquisition, Project administration, Resources, Supervision, Writing – original draft, Writing – review & editing.
